# Naturally induced humoral response against *Plasmodium vivax* reticulocyte binding protein 2P1

**DOI:** 10.1186/s12936-021-03784-1

**Published:** 2021-06-03

**Authors:** Jenni Hietanen, Anongruk Chim-ong, Jetsumon Sattabongkot, Wang Nguitragool

**Affiliations:** 1 Department of Molecular Tropical Medicine and Genetics, Faculty of Tropical Medicine, Mahidol University, 420/6 Ratchawithi Road, Ratchathewi, 10400 Bangkok, Thailand; 2 Mahidol Vivax Research Unit, Faculty of Tropical Medicine, Mahidol University, 420/6 Ratchawithi Road, Ratchathewi, 10400 Bangkok, Thailand

**Keywords:** *Plasmodium vivax*, Malaria, Cytophilic, Complement, Antibody, Serology

## Abstract

**Background:**

*Plasmodium vivax* is the most prevalent malaria parasite in many countries. A better understanding of human immunity to this parasite can provide new insights for vaccine development. *Plasmodium vivax* Reticulocyte Binding Proteins (RBPs) are key parasite proteins that interact with human proteins during erythrocyte invasion and are targets of the human immune response. The aim of this study is to characterize the human antibody response to RBP2P1, the most recently described member of the RBP family.

**Methods:**

The levels of total IgG and IgM against RBP2P1 were measured using plasmas from 68 *P. vivax* malaria patients and 525 villagers in a malarious village of western Thailand. The latter group comprises asymptomatic carriers and healthy uninfected individuals. Subsets of plasma samples were evaluated for anti-RBP2P1 IgG subtypes and complement-fixing activity.

**Results:**

As age increased, it was found that the level of anti-RBP2P1 IgG increased while the level of IgM decreased. The main anti-RBP2P1 IgG subtypes were IgG1 and IgG3. The IgG3-seropositive rate was higher in asymptomatic carriers than in patients. The higher level of IgG3 was correlated with higher *in vitro* RBP2P1-mediated complement fixing activity.

**Conclusions:**

In natural infection, the primary IgG response to RBP2P1 was IgG1 and IgG3. The predominance of these cytophilic subtypes and the elevated level of IgG3 correlating with complement fixing activity, suggest a possible role of anti-RBP2P1 antibodies in immunity against *P. vivax*.

## Background

*Plasmodium vivax* malaria remains a major public health problem in many countries. At present, there is no approved vaccine for *P. vivax*, but such a vaccine would be highly useful for the global malaria eradication. Several types of vaccines have been considered for *P. vivax* malaria, including one to prevent or eliminate liver stage infection (pre-erythrocytic vaccine), one to reduce blood stage parasitaemia (blood stage vaccine), and one to block transmission from humans to mosquitoes (transmission-blocking vaccine). Blood stage vaccines are aimed to neutralize red cell infection, which is the immediate cause of malaria symptoms. Blood stage vaccines for *P. vivax* will help reduce disease transmission because the density of gametocytes, the stage transmissible to the mosquitoes, is closely linked to the total blood parasitaemia [1–3]. Several *P. vivax* asexual blood stage antigens have been considered for vaccine development, including Duffy binding protein (DBP) [4], several Reticulocyte binding proteins (RBPs) [5], apical membrane antigen 1 (AMA1), and merozoite surface proteins (MSPs) [6]. Currently the most advanced candidate is the *P. vivax* Duffy Binding Protein (PvDBP), which has entered Phase 1a clinical trials [7, 8]. Other targets [5, 8] are much further behind and new candidates are still needed to maintain a healthy vaccine development pipeline.

*Plasmodium vivax* RBPs are a major group of *P. vivax* invasion ligands. Human antibodies to some of these proteins have been shown to be associated with clinical protection [9–11]. Antibodies to one of them, RBP2b, directly inhibit erythrocyte invasion [12]. Recently, we characterized a novel RBP, RBP2P1, and found that a higher level of total IgG to RBP2P1 is associated with lower parasitaemia, suggesting an involvement in functional immunity [13]. However, the analysis was limited to total IgG.

This study aims to provide a more complete account of human antibody response to RBP2P1. Plasmas from acute *P. vivax* malaria patients and the general population in an endemic area in Thailand were examined for IgM, total IgG, IgG subtypes, and anti-RBP2P1 antibody-mediated complement fixing activity.

## Methods

The use of human specimens in this study was approved by the Ethics Committee of the Faculty of Tropical Medicine, Mahidol University.

### Study sites

Malaria transmission in Thailand is seasonal and found mainly near the country border, with Myanmar to the west, Cambodia to the east and Malaysia to the south [14–16]. The study sites, Kanchanaburi and Ratchaburi provinces, are located on the western border. Both provinces are endemic for malaria [3]. At the time of sample collection (September-October 2012), the study sites had an overall malaria prevalence of 4.18 % by PCR (*P. vivax* 3.09 %, *Plasmodium falciparum* 0.86 % and mixed *P. vivax*/ *P. falciparum* 0.26 %) [3]. In a cohort study conducted shortly thereafter (2013–2014) [17], the prevalence varied seasonally from 1.7 to 4.2 % for *P. vivax* and 0–1.3 % for *P. falciparum*. The infections were found primarily in a small number of individuals who were positive at multiple time points during the monthly active surveys. Most infections (90 %) were asymptomatic, as confirmed by the lack of malaria-like symptoms during the follow-up period.

### Study specimens

During the 2012 cross-sectional study [3], plasma samples were collected from the general population and a subset of these plasma samples used in this current study. Among the volunteers, 26 had low-density *P. vivax* infection without concurrent fever (body temperature > 37.5 °C) and no history of fever or feeling unwell within the preceding 48 h. These 26 people were classified as asymptomatic carriers in this study. In addition to these cross-sectional survey samples, additional plasma samples were obtained from 68 *P. vivax* acute malaria patients (PCR-confirmed) from the same village in 2012–2013. The summary of the study population characteristics is provided in Table 1. Seven plasma samples from unknown healthy donors from Bangkok, a non-endemic area, were obtained from the Thai Red Cross and used as the negative control for the antibody-typing assays. For the complement assay, additional 8 negative samples were obtained from the Thai Red Cross, making the total number of negative control samples 15.

**Table 1:** Characteristics of the 593 study volunteers from western Thailand

Parameter	Uninfected^a^	Asymptomatic^a^	Patient^b^
n	499	26	68
Male sex, no. (%)	227 (46)	19 (73)	50 (74)
Age, median (range)	20 (0.8–92)	35 (8–70)	29 (18–71)
0–6 years, n	81	–	–
7–12 years, n	98	2	–
13–17 years, n	48	4	–
18 + years, n	272	20	68

^a^ From a cross-sectional malaria survey

^b^ Acute *P. vivax* malaria patients from health facilities

### Expression and purification of PvRBP2-P1

As described earlier [13], the full-length RBP2-P1 (1788 bp, signal peptide excluded) was expressed in *Escherichia coli* SHuffle cells as a soluble protein. The recombinant RBP2P1 protein (70 kDa) was purified by metal affinity chromatography and, after removal of 6-His tag, further purified with FPLC.

### Antibody measurements

Antibody levels were measured by using a Bio-plex (Bio-Rad) bead-based assay as described [18]. Briefly, COOH microspheres (2.5 × 10^6^, Luminex Corp) were washed with PBS (Phosphate Buffered Saline), incubated at room temperature for 20 min at constant agitation with 100 mM monobasic sodium phosphate (pH 6.2), 50 mg/ml sulfo-NHS (N-hydroxysulfosuccinimide sodium salt) and 50 mg/ml of EDC [N-(3-dimethylaminopropyl)-N′-ethylcarbodiimide hydrochloride] to activate the amine groups on the microspheres’ surface to capture the carboxyl groups of the target protein. Coupling reaction was incubated overnight at 4 °C with constant agitation. On the next day microspheres were washed with PBS-TBN (1X PBS buffer pH 7.4, with 0.05 % Tween 20. 1 % BSA, 0.1 % Sodium Azide) buffer and stored in the same buffer until antibody measurements. Protein concentration used for the coupling was optimized by experimentally testing the amount of protein that would generate a log-linear standard curve using a positive control plasma pool prepared from Thai *P. vivax* patient samples.

Plasma samples were diluted 1/100 when measuring IgG and 1/200 for IgM detection in PBS with 1 % BSA and 0.05 % Tween (PBT). Each diluted plasma (50 µl) was added to a 96-well Multiscreen filter plate together with 0.1 µl of coupled RBP2-P1 microspheres in 50 µl of PBT per well. The plate was incubated at room temperature for 30 min on a plate shaker. After incubation, the microspheres were washed three times with 100 µl of PBT. Then, 100 µl of 1/100 dilution in PBT of the following IgG or IgG subtype detector antibodies were used: donkey anti-human IgG Fc-PE (0.5 mg/ml, Jackson ImmunoResearch), mouse anti-human IgG1 hinge-PE (0.1 mg/ml, Southern Biotech), mouse anti-human IgG2 Fc-PE (0.1 mg/ml, Southern Biotech), mouse anti-human IgG3 hinge-PE (0.1 mg/ml, Southern Biotech) and mouse anti-human IgG4 Fc-PE (0.1 mg/ml, Southern Biotech). For IgM detection, 100 µl of 1/400 dilution in PBT of donkey anti-human IgM Fc5u-PE (0.5 mg/ml, Jackson ImmunoResearch) was used. Detector antibodies were incubated with the microspheres at room temperature for 15 min on a plate shaker. Microspheres were washed three times with 100 µl of PBT and resuspended to 100 µl of PBT. Fluorescence was measured with Bio-Plex 200®. Bio-Plex 200® gave the result as the median fluorescent intensity (MFI). Each sample was standardized to the arbitrary unit (AU) by an in-plate standard curve generated by running a two-fold serial dilution (IgG: 1/50 to 1/25,600 and IgM 1/25 to 1/25,600) of the positive control plasma pool from *P. vivax* patients. To determine the fluorescence background, several blank wells without plasma were run in each plate. Plasma from healthy volunteers were included as the negative control for each assay. The seropositivity threshold was set as the mean + 2 standard deviations (SD) of the negative controls.

### Complement-fixation assay

The complement fixation potential of anti-RBP2P1 antibodies were measured by ELISA-based assay as described earlier [19]. Briefly, Nunc Maxisorp 96-well plates were coated with 10 µg/ml of PvRBP2-P1 and incubated overnight at 4 °C. At each step the volume added per well was 100 µl. Wells were blocked with 10 % skim milk in PBS-Tween (1x PBS + 0.01 % Tween 20) for 1 h. After blocking, wells were washed three times with PBS-Tween. Plasma samples were heat-inactivated at 56 °C for 30 min to destroy the complement. After that, samples were diluted 1/100 in 1 % skim milk in PBS-Tween and were added to the plate and incubated at RT for 1 h. Plates were washed three times with PBS-Tween. Human complement protein C1q (Merck Millipore) in 1 % skim milk and PBS-Tween was added at 10 µg/ml and incubated at RT for 30 min. Plates were washed three times with PBS-Tween. 1/2000 in 1 % skim milk and PBS-Tween diluted chicken anti-human C1q antibody (Merck Sigma-Aldrich) was then added and incubated 1 h at RT. Wells were washed three times with PBS-Tween. To detect bound C1q, 1/4000 in 1 % skim milk and PBS-Tween diluted rabbit anti-chicken-IgY-(IgG)-HRP (Merck Sigma-Aldrich) was added and incubated for 1 h. Wells were washed three times with PBS-Tween and then finally three times with PBS. Detection of C1q fixation was done by incubating with ABTS (Merck Millipore) for 30 min and absorbance quantified by using a plate reader. OD values were normalized against a positive control sample (a *P. vivax* patient plasma) and reported as AU. This control sample was used on all plates to permit comparison of data across different plates.

### Statistical analysis

MFI values were converted to relative antibody units. Further analysis and data presentation was performed in Prism version 6 (GraphPad, USA) or PASW Statistics version 18.0.0. Differences in the antibody levels between groups of different infection status were compared by using the Kruskal-Wallis test with Dunn’s multiple-comparison test and data are presented using box-plots, with error bars showing the 5–95 percentile range and dots representing the outliers. Differences in seroprevalence between age groups or groups of different infection status was tested with the Fisher’s exact test. Spearman’s rank correlation was used to determine correlation between parameters as specified in the text.

## Results

### IgM and total IgG in the community survey

**Fig. 1:**
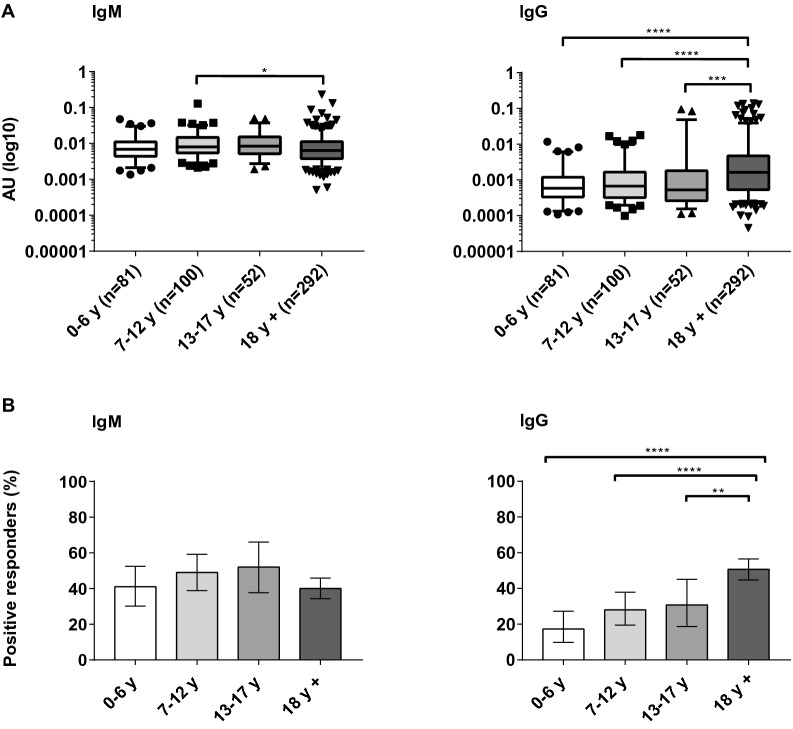
IgM and IgG responses in a cross-sectional survey: **A** Antibody levels. Box-plots represent the median and the interquartile range; error bars indicate the 5–95 percentiles; filled symbols represent outliers. Differences in the antibody levels between the age groups were analyzed with Kruskal-Wallis test with Dunn’s multiple-comparison test. **B** Seropositive rates. Bars represent the proportions of IgM and IgG seropositive individuals in the different age groups. The seropositive cut off values (mean + 2SD) for antibody types were: IgM 8.82 × 10^− 3^ and IgG 9.32 × 10^− 4^. Error bars indicate 95 % confidence intervals. Differences between the groups were analyzed with Fisher’s exact test. ****p < 0.0001, ***p < 0.001, **p < 0.01, *p < 0.05

525 plasma samples from a cross-sectional survey in Kanchanaburi province were used to examine the presence of RBP2P1 antibodies in the community. The age of the study participants showed a positive correlation with total IgG (Spearman ρ = 0.317, p = 0.01), but a negative correlation with IgM (Spearman ρ = − 0.144, p = 0.001). When the cross-sectional samples were classified into four age groups: 0–6 years, 7–12 years, 13–17 years and ≥ 18 years, the anti-RBP2P1 IgM levels were higher (Kruskal-Wallis test with Dunn’s multiple-comparison test, p = 0.0168) in children aged 7–12 years compared to adults (Fig. 1A). The IgG response (Fig. 1A) was higher in the adult group compared with all younger groups (p < 0.001). Consistently, IgG seropositivity in the adult group was higher compared to the other three groups (Fisher’s exact test, p < 0.01 for all pairwise comparison) (Fig. 1B). There were no significant differences among the age groups for IgM seropositivity (Fisher’s exact test, p > 0.05 for all pairwise comparison) (Fig. 1B).

### Comparison between ***P. vivax*** asymptomatic carriers, patients, and uninfected individuals

34 *P. vivax* malaria patients and 31 selected individuals from the cross-sectional survey (22 asymptomatic *P. vivax* carriers + 9 healthy parasite-free villagers who reported never to have had malaria) were selected for further comparative analysis. The patient specimens were obtained from the malaria clinic in the study village at roughly the same time period (2012–2013). The plasma samples were tested for anti-RBP2P1 IgM, total IgG, IgG1, IgG2, IgG3 and IgG4.

**Fig. 2:**
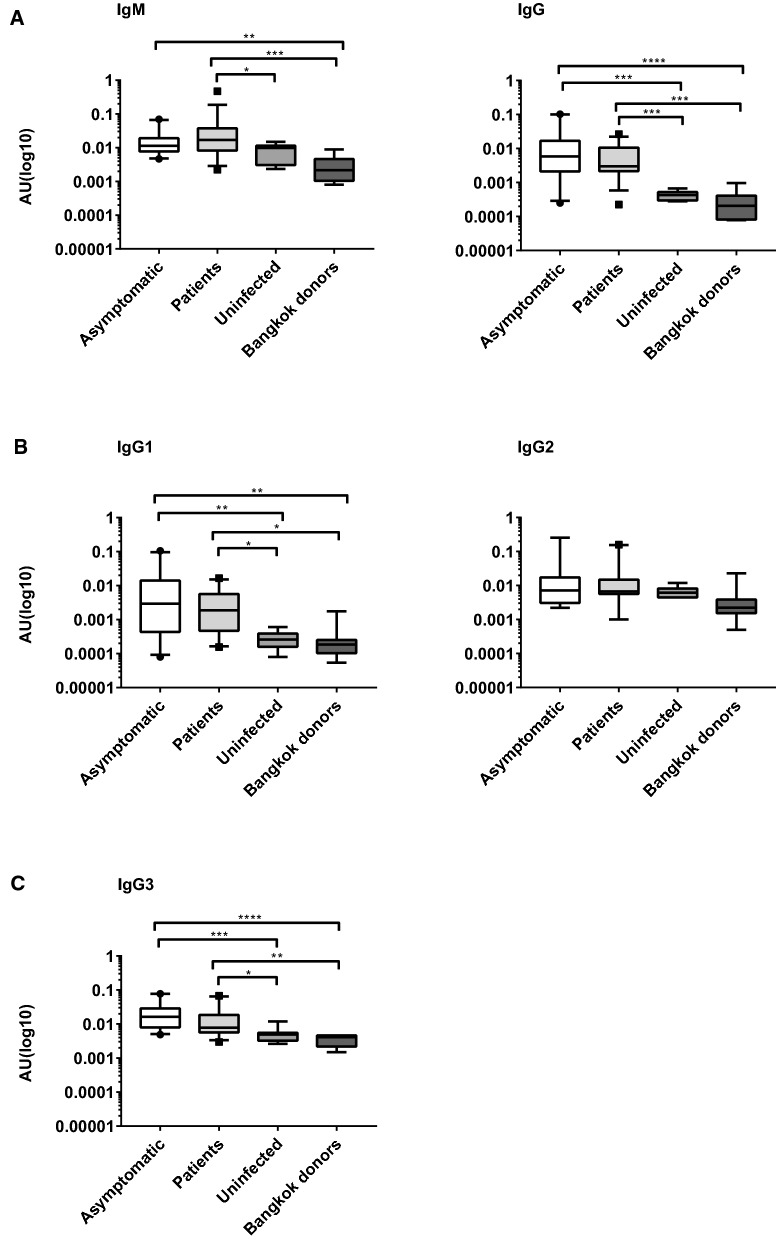
Anti-RBP2P1 IgM, IgG and IgG subtype responses in different types of donors. **A** IgM and IgG responses of a subset of the cross-sectional survey samples (asymptomatic carriers and uninfected villagers), patients and Bangkok donors. **B** IgG1, IgG2 and IgG3 responses in the same four groups. Box-plots represent the median and the interquartile range; error bars indicate the 5–95 percentiles; filled symbols represent outliers. ****p < 0.0001, ***p < 0.001, **p < 0.01, *p < 0.05 (Kruskal-Wallis test with Dunn’s multiple-comparisons test)

The median of anti-RBP2P1 IgM levels in *P. vivax* patients did not differ from that of asymptomatic carriers, but it was higher than that of uninfected villagers or healthy Bangkok donors (Fig. 2A). A similar pattern was found for total IgG (Fig. 2A) as well as IgG1 and IgG3 (Fig. 2B). No statistically significant difference was detected between the four groups for IgG2 (Fig. 2B). IgG4 was not detected in any sample.

**Fig. 3:**
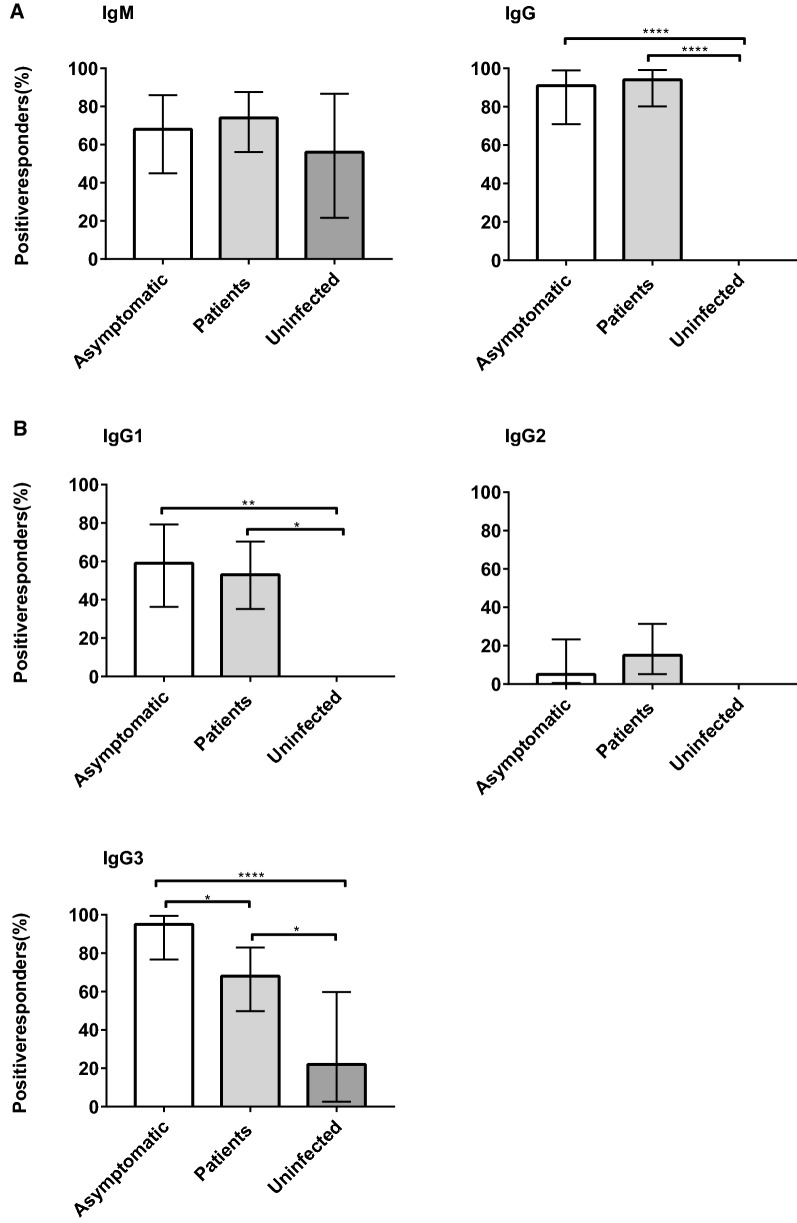
Anti-RBP2P1 seropositivity rates in different types of villagers. **A** IgM and IgG seropositivity rates in *P. vivax* asymptomatic carriers, patients, and uninfected villagers. The seropositive cut off values (mean + 2SD) for antibody types were: IgM 8.82 × 10^− 3^ and IgG 9.32 × 10^− 4^. **B** IgG1, IgG2 and IgG3 seropositivity rates in the same three groups. The seropositive cut off values for antibody types were: IgG1 1.60 × 10^− 3^, IgG2 2.01 × 10^− 2^ and IgG3 4.89 × 10^− 3^. Error bars indicate the 95 % confidence intervals. ****p < 0.0001, ***p < 0.001, **p < 0.01, *p < 0.05 (Fisher’s exact test)

The anti-RBP2P1 seropositive rates in asymptomatic carriers, patients, and uninfected villagers were also compared. The mean + 2SD value of the Bangkok donors was used as the seropositivity threshold. There was no difference in the seropositive rates between the three groups for IgM (Fig. 3A). For total IgG, the seropositive rates were similar between the patients (94 %) and the asymptomatic carriers (91 %). Both groups had a higher seropositive rate than the uninfected villagers did (0 %) (Fisher`s exact test, p < 0.0001) (Fig. 3A). IgG1 seropositivity followed a similar trend (patients 44 % and asymptomatic carriers 59 %), albeit at lower rates in the two infected groups (Fig. 3B). For IgG2, the seropositive rates were low and indistinguishable in all three groups. For IgG3, the seropositive rate was higher in the asymptomatic carriers (95 %) compared to the patients (68 %) (Fisher exact test, p = 0.0183), who in turn, had a higher seropositive rate than the uninfected villagers (22 %) (Fisher’s exact test, p = 0.0226) (Fig. 3B).

### RBP2P1-mediated complement fixation

**Fig. 4:**
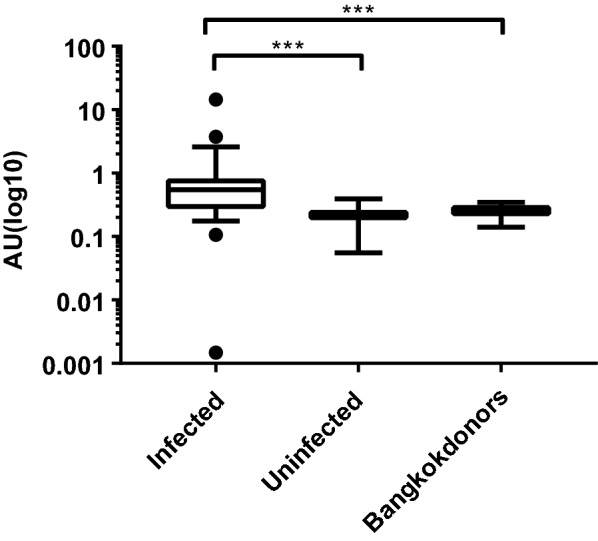
Complement-fixing (C1q-binding) activity of different types of donor plasmas. Box-plots represent the median and the interquartile range; error bars indicate the 5–95 percentiles; filled symbols represent outliers. Filled symbols represent outliers. ****p < 0.0001, ***p < 0.001, **p < 0.01, *p < 0.05 (Kruskal-Wallis test with Dunn’s multiple-comparisons test)

The complement-fixing activities of anti-RBP2P1 antibodies were assessed. The same subpopulation of 22 asymptomatic carriers, 34 patients, and 9 uninfected villagers used for IgG subtyping were subjected to an ELISA-based complement fixation assay (Fig. 4). A set of 15 healthy Bangkok donors were also used as the control. This assay infers the complement fixing activity from the ability of anti-RBP2P1 antibodies to bind human C1q, the first step of the classical complement pathway. Because the asymptomatic and patient groups have indistinguishable complement-fixing activity (data not shown), they were combined into a single *P. vivax* ‘infected’ group (Fig. 4). This group had significantly higher level of complement-fixing activity than the uninfected villagers and the Bangkok donors (Kruskal-Wallis test with Dunn’s multiple-comparison test, p = 0.0001 and p = 0.0002, respectively) (Fig. 4).

**Fig. 5:**
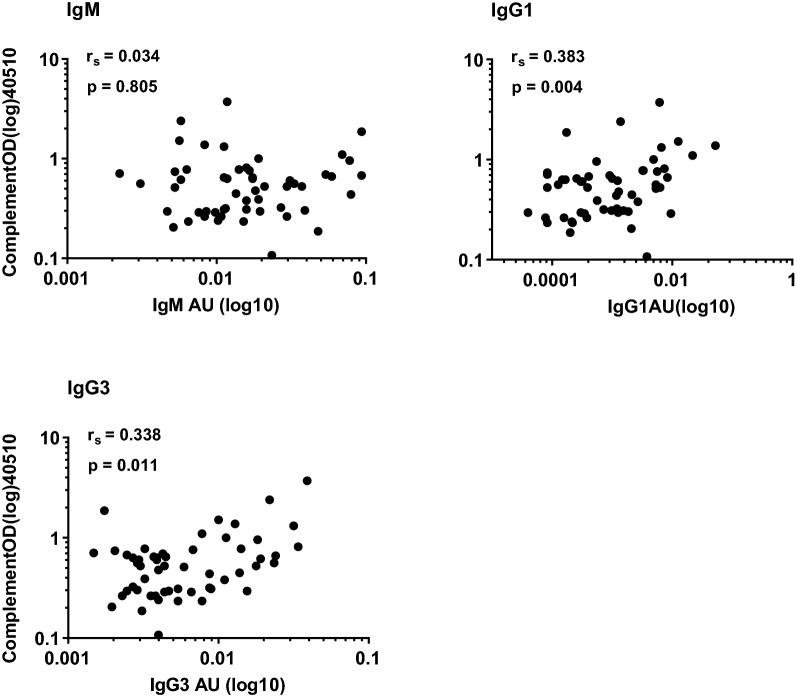
Complement-fixing activity correlates with the IgG1 and IgG3 levels. Antibody levels in arbitrary units (AU) of infected individuals are plotted as a function of complement-fixing (C1q-binding) activity (n = 56). r_s_, Spearman’s rank correlation

To test whether the higher complement-fixing activity in the *P. vivax* infected individuals (n = 56) is associated with IgM and cytophilic antibodies, correlation analysis was performed (Fig. 5). The results show that the levels of IgG1 and IgG3 subtypes, but not IgM, were rank-correlated with complement-fixing activity (Spearman’s rank correlation, p ≤ 0.011). In a multivariate linear regression model using IgM, IgG1, and IgG3 as independent variables, only IgG3 remains significantly associated with complement-fixing activity (p = 0.018).

## Discussion

Naturally acquired antibodies are important factors in protective immunity against malaria [20, 21]. Many merozoite antigens are natural immunogens and actively being pursued as vaccine targets. For *P. falciparum* blood-stage vaccines, several targets, including AMA1, EBA175, GLURP, MSP1, MSP2 and MSP3, have reached clinical trials, and more recent candidates such as reticulocyte-binding protein homolog 5 (PfRh5) are under active research [7, 22]. For the blood stage *P. vivax* vaccine, DBP has been the sole intensively studied candidate [7]. Over the past five years *P. vivax* RBPs have garnered increasing attention as functional invasion ligands of the parasite, markers of exposure, and vaccines candidates [5, 12, 23, 24]. Two members of the family, RBP1a and RBP2b, appear promising for further vaccine development [5]. RBP2P1 is the most recently characterized member of the RBP family [13]. Its small size of 72 kDa allows the nearly full-length recombinant protein expression [13]. Compared to the other members, the antibody response to RBP2P1 has been less well investigated.

In this study, the naturally acquired antibody responses to RBP2P1 among villagers living in a *P. vivax* endemic area of western Thailand were measured. Although the transmission intensity in the study site in Thailand was low (3.1 % *P. vivax* prevalence by qPCR), several villagers still had protective immunity as they could carry the parasite without becoming sick [3, 17]. In this population, the IgM response showed a weak negative correlation with age. In addition, IgG tended to increase with age, similar to trends observed with many *P. vivax* antigens [18, 25–27]. These correlations presumably reflect the maturation of the host immune system from the IgM to the more specific IgG, after IgG memory being boosted over time by repeated exposures [28, 29].

In the adult group, only 7 % had ongoing *P. vivax* infection yet nearly half (48 %) were seropositive for RBP2P1 (Fig. 1C, D). Although the majority of *P. vivax* antigens were reported to have a half-life of less than 6 months [23], there is evidence of long-living antibodies, with PvMSP1 lasting from a year to 30 years and PvMSP8 from 8 to 12 years [30–35]. According to the antibody half-life model from Longley and coworkers who measured the antibody half-lives of over 300 *P. vivax* antigens, the estimated half-life of RBP2P1 antibodies is fairly long at 308 (95 % CI, 218–521) days [24]. Thus, in addition to recent exposure, the long half-life may contribute to the high anti-RBP2P1 IgG seropositivity rate among the uninfected villagers.

The major IgG subtypes reactive to RBP2P1 were IgG1 and IgG3. This is similar to an earlier report for PvRBP1a and PvDBP [11]. The function of cytophilic antibody subtypes IgG1 and IgG3 may extend beyond interfering with red blood cell binding [13, 20] to encompass other effectors functions, such as opsonic phagocytosis [36], antibody-dependent cellular inhibition [37], and complement activation through binding to the C1q protein complex [38]. Between the two cytophilic subtypes, IgG3 is known to have a higher complement fixing potential [38]. We found that the IgG3 seropositivity was higher in asymptomatic carriers than in patients, paralleling the ability of the former groups to control the parasitaemia at a very low level.

Previous studies have reported similar linkage between elevated IgG3 levels against merozoite antigens and protection from clinical symptoms, such as for *P. falciparum* MSP2, MSP3, AMA1, GLURP, EBA175 and *P. vivax* DBP, MSP1 and GAMA [39–43]. However, due to the nature of the cross-sectional study, it remains inconclusive whether IgG3 is responsible for the clinical protection. An analysis of a cohort from an endemic area would provide a clearer answer.

It has become evident that the optimal antigen to be used for malaria vaccine development should not solely trigger the maximum antibody titers, but that these antibodies should also have a function [38]. New tests are needed to assess antibody’s functional properties because the traditional growth inhibition assay does not always predict the protection gained by natural infection or vaccine induced immunity [8, 19]. Additional assays are particularly important with *P. vivax*, which cannot be cultured, making it challenging to test most interventions. The recently developed complement-fixation assay is a useful tool to test whether antibodies against malaria antigens are able to fix the complement, leading to complement-mediated killing of the parasites [19]. Several *P. falciparum* antigens have been identified as targets of complement-fixing antibodies [19, 44]. Of particular interest is circumsporozoite protein PfCSP, the most prominent malaria vaccine target. The major antibody types targeting PfCSP were IgM and cytophilic antibodies IgG1 and IgG3, and they all were able to fix complement in the classical pathway [44]. Only one *P. vivax* antigen, Merozoite Surface Protein 3α (PvMSP3α), has been subjected to the complement-fixing assay. RBP2P1 is the second target evaluated [45]. With PvMSP3α both cytophilic subtypes, IgG1 and IgG3 as well as IgM showed correlation with complement fixation similar to PfCSP [45]. For RBP2P1, IgG3 has the most robust correlation with complement fixation.

## Conclusions

The naturally-acquired humoral immune response against *P. vivax* merozoite protein RBP2P1 is biased towards cytophilic antibodies IgG1 and IgG3. The IgG3 seropositivity rate against RBP2P1 was higher in asymptomatic *P. vivax* carriers than in clinical *P. vivax* malaria patients. The level of IgG3 antibody subtype was correlated with complement fixation activity, suggesting that RBP2P1 is a target of functional human immune response to *P. vivax* infection.

## Data Availability

The datasets used and/or analyzed during the current study are available from the corresponding author on reasonable request.

## Abbreviations

RBP: Reticulocyte binding protein.
Sulfo-NHS: *N*-hydroxysulfosuccinimide sodium salt.
EDC: *N*-(3-dimethylaminopropyl)-*N*′-ethylcarbodiimide hydrochloride.
FPLC: Fast protein liquid chromatography.
PBT: Phosphate buffered saline containing 1 % bovine serum albumin and 0.05 % (v/v) Tween-20.
MFI: Median fluorescent intensity
